# Psychotropic drug use among older Swedish nursing home residents with cognitive impairment and behavioral and psychological symptoms: a cross-sectional questionnaire survey

**DOI:** 10.1186/s12877-026-08014-4

**Published:** 2026-07-22

**Authors:** Eva Sönnerstam, Tomas Andersson, Annica Backman, Anders Sköldunger, David Edvardsson, Maria Gustafsson, Hugo Lövheim

**Affiliations:** 1 Department of Medical and Translational Biology, Umeå University, 901 87 Umeå, Sweden; 2 Department of Community Medicine and Rehabilitation, Umeå University, 901 87 Umeå, Sweden; 3 Department of Nursing, Umeå University, 901 87 Umeå, Sweden; 4 Department of Nursing, Swinburne University of Technology, Melbourne, Australia; 5 Sahlgrenska Academy, Institute of Health and Care Sciences, University of Gothenburg, Gothenburg, Sweden

**Keywords:** Psychotropic drugs, Cognitive impairment, Behavioral and psychological symptoms, Swedish nursing homes, Older residents

## Abstract

**Objectives:**

The aim of the present study was to describe the association between behavioral and psychological symptoms and psychotropic drug use among older Swedish nursing home residents with cognitive impairment.

**Methods:**

Data was collected from a questionnaire survey which was sent out between 4 and 12 February 2019 within the Swedish National Inventory of Care and Health in Residential Aged Care (SWENIS) II. Sixty municipalities in Sweden were randomly selected and invited. Of these, 49 municipalities accepted the invitation. Six withdrew resulting in 43 participating municipalities and 187 nursing homes. The final study population comprised 1,541 participants, 65 years or older, with cognitive impairment. The Swedish Prescribed drug register was utilized to identify psychotropic drug use. Backward eliminating binary logistic regression analyses were conducted.

**Results:**

At least one psychotropic drug was used of 80.8% of the study population, 20.5% used at least one antipsychotic drug, 32.0% used anxiolytic drugs, 21.3% used hypnotic and sedatives, 58.4% used antidepressants and 32.1% of the residents used antidementia drugs. Among residents with mild cognitive impairment, anxiolytics were significantly associated with anxiety. Also, anxiolytics and hypnotics and sedatives were significantly associated with sleep and nighttime behavior disorders. Antidepressants were significantly associated with depression/dysphoria. The same pattern was found among residents with moderate cognitive impairment. However, anxiolytics were also significantly associated with worsening depression/dysphoria. Hypnotics and sedatives were also associated with anxiety and hallucinations. Moreover, antipsychotic drug use was significantly associated with agitation/aggression and anxiety. Among residents with severe cognitive impairment, antipsychotics, anxiolytics and hypnotics and sedatives were significantly associated with aberrant motor behavior. Anxiolytics were also significantly associated with anxiety and apathy/indifference, and hypnotics and sedatives were significantly associated with agitation/aggression and depression/dysphoria.

**Conclusions:**

Psychotropic drug use is remarkably high among Swedish nursing home residents but the prevalence of antidementia drugs is somewhat low. Associations found, call for further studies examining the treatment of behavioral and psychological symptoms (BPS) in this vulnerable group of people.

**Supplementary Information:**

The online version contains supplementary material available at 10.1186/s12877-026-08014-4.

## Introduction

The human population is growing and ageing rapidly [1]. Accordingly, people living with age-related disorders such as major neurocognitive disorder (MND), previously referred to as dementia, will drastically increase in the years to come [2, 3].

MND stems from several underlying pathologies and the clinical presentation varies [4]. However, a common feature of MND is progressive loss of cognitive functions leading to disability [4] and increased care-giver dependence [5, 6]. Over the course of MND, behavioral and psychological symptoms (BPS) are common [7, 8]. Approximately 90% of people with MND exhibit some type of BPS [9] and it is a common reason for moving into nursing home [10]. Accordingly, the prevalence of nursing home residents with BPS is also high and prevalence higher than 90% has been reported [11]. Apart from the obvious negative effect BPS has on the afflicted, BPS constitutes a significant cost [12] and is associated with an increased caregiver burden [13, 14] and increased over-all mortality [15].

Non-pharmacological interventions, which encompass a multimodal, individualized treatment plan, are the first-line recommendation in the treatment of BPS [16, 17]. However, various psychotropic drugs, such as antidementia drugs (including cholinesterase inhibitors and memantine), antidepressants, antipsychotics, anxiolytics and hypnotics and sedatives, are often used in the treatment of BPS [16] and it has been reported that 60–80% of nursing home residents use these types of drugs [18, 19]. BPS such as agitation, aggression, delusions and hallucinations are e.g. commonly treated with antipsychotic drugs [20] and it has been reported that 11.5–37,4% of nursing home residents use at least one antipsychotic drug [18, 21–25]. According to Swedish guidelines [26], the use of antipsychotic drugs is considered inappropriate among older people and especially among people with MND since the use of these medications has been associated with severe side effects, e.g. impaired cognition, extrapyramidal symptoms, cerebrovascular events and increased risk of mortality [26–28]. If pharmacological treatment of severe psychotic symptoms or aggression is needed, antipsychotics may be used with caution, at low doses and for short durations while continuously evaluating its effect [26]. Moreover, it has been reported that 15–34,8% of nursing home residents use anxiolytic drugs [18, 22–25, 29] and approximately the same number of patients use hypnotic and sedative medications [18, 23–25, 29]. Short-acting medications from these drug classes may be used based on actual indication, which should be regularly evaluated [26]. Moreover, the drug treatment should only be used for a short period of time [26] due to the risk of side effects, e.g. falls, delirium [30, 31], daytime fatigue and anticholinergic side effects [32, 33].

Also, antidepressant drugs can be used in the treatment of various BPS, e.g. depression, as selective serotonin reuptake inhibitors (SSRIs) are the first-line treatment option that should be recommended among older people [26]. Moreover, since a serotonin deficit is thought to be associated with agitation and aggression [34, 35], SSRIs can be recommended to treat this type of BPS due to less side effects and better tolerance than antipsychotic drugs [34, 36]. Accordingly, the prevalence of antidepressant drug use in nursing home environments is high [18, 22–25, 29]. However, the effect of these drugs among people with MND is debatable [37] and the use of antidepressant drugs has been associated with falls [38, 39]. More specifically, the use of SSRIs can cause hyponatremia [40], prolongation of QT-interval and Torsades de pointes [41, 42]. It is therefore of importance to continuously evaluate the indication and effect of antidepressant drug use among older people with depression, especially among those with major NCD, to minimize the risks [26].

On the contrary, cholinesterase inhibitors and memantine, commonly referred to as antidementia drugs and described as psychoactive drugs, are considered to be more beneficial drug treatment compared to antipsychotic drugs in the treatment of BPS due to less serious side effects [26]. Also, the use of antidementia drugs is associated with decreased risk of aggressive behavior [43], cholinesteraseinhibitors have shown preventable effects on BPS and memantine is recommended to treat severe aggression before using antipsychotic drugs [26]. Moreover, these types of drugs may have a preventable and delaying effect on initiation of antipsychotic and sedative drug treatment [44]. It has been found that treatment with antidementia drugs is significantly more common among people living in a specialized MND unit [24]. However, the prevalence of antidementia drugs among nursing home residents has also been found to be surprisingly low. One study reported that only 0.8% of the residents in specialized MND units used antidementia drugs [25]. Other studies found that antidementia drugs were utilized among 13.7–24.2% of nursing home residents in general [23, 24, 29].

Since psychotropic drug use often is inappropriate and debatable among nursing home residents, it is important to continuously evaluate patterns of drug use among this vulnerable group of people. The present study therefore aimed to describe the prevalence of drug use and association between BPS symptoms and psychotropic drug use among older Swedish nursing home residents with cognitive impairment.

## Method

### Data collection

To describe the prevalence of drug use and association between BPS symptoms and psychotropic drug use among Swedish nursing home residents with cognitive impairment, data were collected from The Swedish National Inventory of Care and Health in Residential Aged Care (SWENIS) II study which was a follow-up study to SWENIS I [45]. SWENIS I and II were cross-sectional survey-based studies with the purpose to explore factors related to health, well-being, activities of daily living (ADL)-functioning and person-centered care in Swedish nursing homes.

In SWENIS I, conducted November 2013 through September 2014, 60 of Sweden’s 290 municipalities were randomly selected and invited for participation in the study. Initially 47 municipal managers agreed to participate, five municipalities did not respond, and five municipalities withdrew resulting in 37 municipalities. All nursing homes within these municipalities were contacted and oral and written information about the study was given to the nursing homes. Of 202 nursing homes in the 37 municipalities, completed surveys were received from nursing homes in 35 municipalities.

In SWENIS II, which was conducted November 2018 through May 2019, the 35 municipalities included in SWENIS I were reinvited. Also, another 25 randomized municipalities were asked to participate to match the design in SWENIS I where 60 municipalities were asked to participate. Initially 49 municipalities accepted. Six of these withdrew leaving a total of 43 municipalities including 187 participating nursing homes. Flow chart and geographical distribution of participating municipalities are shown in Fig. A.1 and A.2 (Additional file 1). The surveys were sent out to the nursing homes between 4 and 12 February 2019. Three reminders were sent and the data collection ended April 2019. The survey questionnaire consisted of 149 items and was based on proxy-ratings made by the nursing-staff member who knew the resident best [45]. Besides demographic data, the survey included measures regarding depression [46], pain [47], participation in activities [45], Health Related Quality of Life (EQ-5D) [48], The Thriving of Older People Assessment Scale [49, 50], The Gottfries’ Cognitive Scale [51], The Neuropsychiatric Inventory – Nursing Home (NPI-NH) [52] and The Katz’ Index of Independence in Activities of Daily Living (Katz’ ADL) [53] of which the latter three measures were assessed in the current study.

### Data assessments

#### Gottfries’ cognitive scale

Cognitive impairment was evaluated using The Gottfries’ scale which was developed by Gottfries and Gottfries and contains 27 items [51]. The scale ranges between 0 and 27 points and has negligible floor effects which makes it appropriate to use in nursing home environments where severe cognitive impairment is common, and floor effects can pose a problem when it comes to evaluating severity of MND [51]. Cut-offs for cognitive impairment have been validated against the more commonly used cognitive test Mini Mental State Examination (MMSE) and scores less than 24, on the 27-point scale, correlates with 90% sensitivity and 91% specificity to the usual cut-off of 24/30 for cognitive impairment in the MMSE [54, 55]. A missing item was scored 0.5 for up to 3 items per individual according to the suggested imputation strategy for the scale [51]. The inclusion criterion in the present study was participants with cognitive impairment, i.e. Gottfries’ score < 24. Cognitive impairment was further categorized into mild (16–23), moderate (8–15) and severe (0–7) cognitive impairment based on total Gottfries’ score.

#### The Neuropsychiatric Inventory – Nursing Home (NPI-NH)

BPS was measured utilizing The Neuropsychiatric Inventory - Nursing Home (NPI-NH) which was developed to characterize neuropsychiatric symptoms and psychopathology among nursing home residents with MND [52]. The inventory is based on proxy ratings from caregivers and divided into 12 items including ten behavioral domains (delusions, hallucinations, agitation/aggression, depression/dysphoria, anxiety, elation/euphoria, apathy/indifference, disinhibition, irritability/lability and aberrant motor behavior) and two neurovegetative changes (sleep and nighttime behavior disorders and appetite and eating disorders). Severity and frequency are scored from 0 to 4 and 0 to 3 respectively with a higher score indicating more severe or frequent symptoms. The severity and frequency scores are then multiplied to produce the final score of each item, and the sum of all items can be utilized as a measure of total BPS load, ranging between 0 and 144. Both individual item scores and the total NPI-NH-score are utilized in the present study.

#### The Katz’ Index of Independence in ADL (the Katz’ ADL)

The Katz’ ADL aims to evaluate the individuals’ ability to cope with bathing, dressing, toileting, transferring, continence and feeding. The categories are scored from independence (1) to dependence (0), where six points indicate full ADL independence [53].

### Definition of psychotropic drug use

Psychotropic drugs were defined according to the Anatomical Therapeutic Chemical (ATC) Classification System as antipsychotics (N05A), anxiolytics (N05B), hypnotics and sedatives (N05C) and antidepressants (N06A). Moreover, antidementia drugs (N06D) were defined as psychotropic drugs within this study even if these types of drugs are rather described as psychoactive drugs. Due to the different recommendations and their different effect on BPS, antidementia drugs were further categorized into acetylcholinesterase inhibitors (N06DA) and memantine (N06DX), excluding lecanemab and donanemab as these drugs were not approved when the questionnaire was distributed. Information about psychotropic drug use was collected on ATC code level 7 from the Swedish Prescribed drug register which contains data on every prescribed drug dispensed at pharmacies in Sweden. The register was established in July 2005 and contains data regarding, e.g., the patient, prescription, prescribed drug, prescriber and price [56]. Psychotropic drug use was identified on substance level and further grouped as mentioned above. Drug use was defined as having collected at least one of the specified ATC-codes, included in the psychotropic drug groups, at least once between 1 January and 30 June 2019. The Swedish state subsidizes drug treatment for a maximum of 90 days supply at each refill. Therefore, we chose to identify drug dispensations during a six-month period in the present study.

### Study population

In total, 3,894 surveys returned resulting in a response rate of 55%. Surveys with missing Swedish personal identity number (*n* = 1,123), participants who died before the 30th of June (*n* = 292), without cognitive impairment, i.e. Gottfries’ score ≥ 24 (*n* = 780), surveys with missing values on the Gottfries’ scale (*n* = 37) and residents younger than 65 years (*n* = 121) were excluded. Accordingly, the final study population comprised 1,541 participants. Flow chart is shown in Fig. A.1 (Additional file 1).

### Statistics

Basic characteristics of the study population are presented as frequencies for categorical variables (degree of cognitive impairment and sex), mean values with standard deviation (SD) for continuous variables with normal distribution (age, Gottfries’ score and ADL) and median values with interquartile range (IQR) for continuous variables with skewed distribution (total NPI-NH score).

Psychotropic drug use is presented as frequencies. Chi-2-tests were conducted to compare the use of different drug classes between the residents with different levels of cognitive impairment. The association between BPS and psychotropic drug use was investigated using backward eliminating binary logistic regression models, stratified for mild, moderate and severe cognitive impairment. Separate analyses were conducted with drug use, according to each ATC-group (e.g. N05A), as the dependent variable and NPI-NH score for each item (e.g. NPI-NH delusion score) as the independent variable. Each analysis was adjusted for sex (entered as a dichotomous variable), age, Gottfries’ cognitive score, ADL and the other NPI-NH item scores (all entered as continuous variables). Results are presented as odds-ratio (OR) with 95% confidence interval (CI). A p-value of < 0.05 was considered statistically significant.

## Results

Basic characteristics of the study population are shown in Table 1. Mild and moderate cognitive impairment were more common than severe cognitive impairment in the study population. The mean age and range were approximately the same regardless of cognitive function. Almost two thirds were women among those with mild and moderate cognitive impairment. Among those with severe cognitive impairment, the frequency of women was higher; 76.5% (n = 260). Residents with severe cognitive impairment had the lowest mean Gottfries’ and Katz’ ADL score; 4.0 (± 2.2) and 1.1 (± 1.4), respectively. Among residents with moderate and severe cognitive impairment the median NPI-NH score was 12.0 (6.0–25.0) and 13.0 (5.0–26.0), respectively.

**Table 1:** Basic characteristics of the study population categorized according to level of cognitive impairment

Level of cognitive impairment, *n* (%)	Mild^a^, 620 (40.2)	Moderate^b^, 581 (37.7)	Severe^c^, 340 (22.1)	Total, 1,541 (100.0)
Age, mean ± SD (range)	85.8 ± 7.7 (65–106)	85.9 ± 7.3 (65–103)	84.6 ± 7.9 (65–105)	85.6 ± 7.6 (65–106)
Women^d^, *n* (%)	408 (65.8)	378 (65.1)	260 (76.5)	1,046 (67.9)
Gottfries’ score (0–27), mean ± SD	19.4 ± 2.3	11.8 ± 2.2	4.0 ± 2.2	13.1 ± 6.3
Total NPI-NH (0–144), median (IQR)	8.5 (3.0–17.0)	12.0 (6.0*–*25.0)	13.0 (5.0*–*26.0)	11.0 (5.0*–*22.0)
Katz’ ADL score (0–6)^d^, mean ± SD	3.5 ± 2.0	2.6 ± 1.9	1.1 ± 1.4	2.7 ± 2.1

*NPI-NH* Neuropsychiatric Inventory Nursing Home, *ADL* Activities of Daily Living

^a^ Gottfries’ score 16–23

^b^ Gottfries score 8–15

^c^ Gottfries score 0–7

^d^ Missing: Sex, *n* = 3; Katz’ ADL score, *n* = 88

Psychotropic drug use was common and 80.8% of the study population used at least one psychotropic drug (Table 2). Approximately three quarters of the residents used at least one antipsychotic, anxiolytic, hypnotic or sedative, or antidepressant drug and the prevalence was approximately the same regardless degree of cognitive impairment. More than half of the residents used at least one antidepressant drug. Accordingly, this was the most commonly used drug class regardless level of cognitive function. Antidementia drugs were the second most commonly used drug class among the residents with higher frequency among those with moderate and severe cognitive impairment, 36.8% (*n* = 214) and 35.3% (*n* = 120), respectively. The same pattern and almost the same prevalences were found for anxiolytic drug use which was the third most commonly used psychotropic drug class. Antipsychotics were the least used drug class among residents with mild and moderate cognitive impairment, 18.9% (*n* = 117) and 20.5% (*n* = 119), respectively. However, more than one fifth of those with severe cognitive impairment used at least one antipsychotic drug. The least used drug class among residents with severe cognitive impairment was hypnotics and sedatives, 17.9% (*n* = 61). On the contrary, more than one fifth used at least one type of hypnotic or sedative drug among those with mild and moderate cognitive impairment.

**Table 2:** Frequency of antipsychotic drug use according to level of cognitive impairment

Psychotropic drug class (ATC-code)^a^	Level of cognitive impairment				
	Total	Mild	Moderate	Severe	*p*-value^*^
Antipsychotics (N05A), *n* (%)	316 (20.5)	117 (18.9)	119 (20.5)	80 (23.5)	0.23
Anxiolytics (N05B), *n* (%)	493 (32.0)	173 (27.9)	210 (36.1)	110 (32.4)	0.01
Hypnotics and sedatives (N05C), *n* (%)	329 (21.3)	146 (23.5)	122 (21.0)	61 (17.9)	0.13
Antidepressants (N06A), *n* (%)	900 (58.4)	374 (60.3)	337 (58.0)	189 (55.6)	0.36
Antidementia drugs (N06D), *n* (%)	494 (32.1)	160 (25.8)	214 (36.8)	120 (35.3)	< 0.001
Acetylcholinesterase inhibitors (N06DA), *n* (%)	262 (17.0)	99 (16.0)	118 (20.3)	45 (13.2)	0.02
Memantine (N06DX), *n* (%)	337 (21.9)	93 (15.0)	141 (24.3)	103 (30.3)	< 0.001
At least one of the psychotropic drugs	1,245 (80.8)	487 (78.5)	484 (83.3)	274 (80.6)	0.11
At least one drug of N05A-C or N06A	1,147 (74.4)	457 (73.7)	438 (75.4)	252 (74.1)	0.79

^a^ Missing: Drug use among mild cognitive impairment (*n* = 3), moderate cognitive impairment (*n* = 2), severe cognitive impairment (*n* = 2)

* *p*-value < 0.05 considered statistically significant

The logistic regression models revealed several statistically significant associations between higher NPI-NH items score and psychotropic drug use. The results are presented for mild, moderate and severe cognitive impairment in Table A.1-A.3 (Additional file 2).

It was found that anxiolytic drug use was significantly associated with aberrant motor behavior (OR 0.90, 95% CI 0.82–0.98) among those with mild cognitive impairment. Moreover, the use of acetylcholinesterase inhibitors was significantly associated with hallucinations (OR 1.11, 95% CI 1.01–1.21) and memantine was significantly associated with experiencing increased delusions (OR 1.13, 95% CI 1.05–1.22) and having appetite and eating disorders (OR 0.82, 95% CI 0.69–0.98). All significant associations found between BPS and basic characteristics and psychotropic drug use are further presented in Table A.1 (Additional file 2).

The use of antipsychotic drugs was significantly associated with increasing anxiety among residents with moderate cognitive impairment (OR 1.07, 95% CI 1.01–1.14). A significant association between the use of hypnotics and sedatives and hallucinations was also found (OR 1.08, 95% CI 1.002–1.16). Both acetylcholinesterase inhibitors and memantine were significantly associated with elevated irritability/lability (OR 1.12, 95% CI 1.05–1.20; OR 1.08, 95% CI 1.02–1.15). Additionally, the use of acetylcholinesterase inhibitors was significantly associated among those with sleep and nighttime behavior disorders (OR 0.89, 95% CI 0.80–0.98). Memantine was significantly associated with symptoms of elation/euphoria (OR 1.14, 95% CI 1.002–1.29). BPS and basic characteristics significantly associated with psychotropic drug use are presented in Table A.2 (Additional file 2).

The use of antipsychotic, hypnotic and sedative and anxiolytic drugs was significantly associated with increasing aberrant motor behavior among residents with severe cognitive impairment (OR 1.13, 95% CI 1.05–1.21; OR 1.19, 95% CI 1.10–1.29; OR 1.13, 95% CI 1.03–1.23). Anxiolytic drug use was also significantly associated with increasing apathy/indifference (OR 1.09, 95% CI 1.007–1.18). Also, hypnotics and sedatives were significantly associated with agitation/aggression (OR 1.12, 95% CI 1.12–1.23). The use of memantine was significantly associated with delusions (OR 0.84, 95% CI 0.74–0.96), hallucinations (OR 1.14, 95% CI 1.03–1.27) and appetite and eating disorders (OR 0.83, 95% CI 0.70-0.996). See Table A.3 (Additional file 2) for significant associations between BPS and basic characteristics, and psychotropic drug use among people with severe cognitive impairment.

Several of the significant associations between BPS and psychotropic drug use among residents with mild, moderate and severe cognitive impairment were also found when investigating associations among the total study population regardless level of cognitive impairment (Table 3). Additionally, it was found that anxiolytic drug use was significantly associated with worsening irritability/lability (OR 1.05, 95% CI 1.01–1.10). The use of antidementia drugs was significantly associated with apathy/indifference (OR 0.94, 95% CI 0.89–0.99). Moreover, an association was found between memantine usage and anxiety (OR 1.05, 95% CI 1.002–1.09). For significant associations between BPS and basic characteristics, and psychotropic drug use, regardless level of cognitive impairment, see Table 3.

**Table 3:** Significant associations between BPS and basic characteristics, and psychotropic drug use among all residents regardless level of cognitive impairment. Results are presented as odds-ratios (OR) with the 95%-confidence interval (CI)

	Antipsychotics	Anxiolytics	Hypnotics and sedatives	Antidepressants	Antidementia drugs	Acetylcholinesterase inhibitors	Memantine
Age	0.95 (0.94–0.97)			0.98 (0.97–0.997)	0.97 (0.96–0.99)	0.95 (0.94–0.97)	0.97 (0.96–0.99)
Gottfries’ score (0–23)			1.03 (1.01–1.05)		0.94 (0.92–0.96)	0.97 (0.95–0.995)	0.92 (0.90–0.95)
Katz’ ADL (0–6)		1.08 (1.01–1.15)			1.22 (1.15–1.30)	1.29 (1.19–1.39)	1.17 (1.09–1.26)
Agitation/ Aggression	1.06 (1.02–1.10)						
Depression/Dysphoria				1.16 (1.10–1.23)			
Anxiety	1.05 (1.01–1.10)	1.11 (1.06–1.16)	1.07 (1.03–1.12)				1.05 (1.002–1.09)
Apathy/ Indifference					0.94 (0.89–0.99)		
Irritability/ Lability		1.05 (1.01–1.10)			1.07 (1.02–1.11)		1.06 (1.02–1.11)
Sleep and Nighttime Behavior Disorders		1.14 (1.09–1.20)	1.17 (1.11–1.23)				
Appetite and Eating Disorders					0.93 (0.88–0.99)		0.90 (0.83–0.97)

*ADL * Activities of Daily Living

## Discussion

Despite the risk of side effects and the weak evidence-based support for treatment with psychotropic drugs it was found that this type of drug use was high in the total study population, i.e. 80.8% used at least one psychotropic drug. This adds to the evidence from previous Swedish studies [43, 57]. A possible explanation is that a common reason for admittance to nursing homes in Sweden is severe BPS [10], which are challenging to handle in the regular home environment, resulting in a study population with psychiatric symptoms requiring psychotropic drug treatment.

A high proportion, 20.5%, of the residents used at least one antipsychotic drug, a result similar to a previous study conducted in Norway, which reported a prevalence of 20% [23]. The result is however lower compared to other studies conducted in the Netherlands 2018 and ten years earlier, which found that 25-37.4% of the residents used antipsychotic drugs [18, 22, 25]. Nevertheless, the prevalence is higher compared with a Swedish study based on data from 2013 which reported a prevalence of 16.0% [58] and in line or somewhat lower compared with a recently published Swedish nation-wide study based on data from 2019 where it was found that 23.5% used at least one antipsychotic drug [59]. Most concerning is that the highest prevalence is found among those with severe cognitive impairment. Antipsychotics should be used with caution, primarily for treating psychotic symptoms or severe aggression [26]. In light of this, the association between antipsychotic drug treatment and agitation/aggression found in the present study, is in accordance with previous studies [21, 22] and could indicate a sound prescription behavior. Also, antipsychotic drug use was significantly lower among those at older age regardless level of cognitive impairment indicating an increased awareness of the severe risks of this type of drug treatment among older people and especially among older people with major NCD. More specifically, the results indicate that careful considerations are made when prescribing these types of drugs among older people living in nursing homes, leading to decreased prescribing as age increases in accordance with recommendations in Swedish guidelines [26]. No significant association was however found between antipsychotics and psychotic symptoms which is in contrast to other results [18, 21]. Instead, antipsychotic drugs were significantly more common among those with anxiety, which is a new finding. Also, antipsychotic drug use was significantly more common among those with severe cognitive impairment exhibiting aberrant motor behavior which has been reported in other studies as well [18, 21]. This association is alarming since it has been found that environmental factors such as level of staff distress due to the patients’ symptoms or physician availability per patient affect the prescription of antipsychotics [22]. Additionally, anxiolytics, hypnotics and sedatives were significantly more common among people with severe cognitive impairment and aberrant motor behavior. Also, hypnotics and sedatives were significantly more common among residents with moderate cognitive impairment exhibiting hallucinations, and those with severe cognitive impairment with agitation and aggression. Altogether, this indicates irrational off-label prescribing among the most vulnerable group of people.

The prevalence of anxiolytics and hypnotics and sedatives is also high in the present study. Even if the prevalence is in accordance with other studies [18, 24, 25, 29], it is worrying due to the known adverse drug reactions associated with this type of drug treatment. Nevertheless, the result also indicates a rational prescribing pattern as anxiolytics, hypnotics and sedatives were significantly more common among those with anxiety and sleep and nighttime behavior. These associations have been found in previous studies as well, which supports the result in the present study [18, 22, 25]. Insomnia symptoms are common among people with cognitive impairment [60]. Additionally, the association between hypnotics and sedatives and sleep and nighttime behavior was only present among those with mild and moderate cognitive impairment. This result seems reasonable as sleep and nighttime behaviors appear to decline among those with severe cognitive impairment [60] among whom the lowest prevalence of hypnotics and sedatives was present.

Antidepressants are one of the most commonly prescribed psychotropic drugs among nursing home residents [18, 22–25, 29] as depression and anxiety are common among older people [61, 62] and especially among those with MND [63, 64]. A positive association was also found between antidepressants and depression/dysphoria in the present study, which indicates that these drugs are used for the right indication. It is questionable though, that no associations were found between antidepressants and agitation and aggression. Aggressive behavior has been found to be more common among those also exhibiting dysphoric symptoms [46] and SSRIs are preferable to treat these types of BPS [34, 36]. However, it can be viewed the other way around, i.e. that aggression and agitation are managed due to treatment with SSRIs. Altogether, this might explain that more than every second nursing home resident used some type of antidepressant drug.

Given that the study population comprised nursing home residents with cognitive impairment, the prevalence of antidementia drugs is somewhat low, even taking into account that a proportion of the individuals have MND of other causes than either Alzheimer’s disease, Lewy-body disease, or Parkinson’s disease with dementia, for which these drugs are used. A possible explanation could be a high number of persons with undiagnosed MND in nursing home environments, as presented in a previous study [65]. Moreover, the low prevalence may also be due to deprescribing among residents that have already tried antidementia drugs and do not respond to these types of drugs anymore due to the MND progression. Also, side effects among some of the residents may have led to deprescribing of antidementia drugs. Few studies have previously looked at associations between antidementia drugs and BPS. In contrast to the other psychotropic drugs investigated in this study, the association between antidementia drugs and BPS was diverse. This may be due to the individual manifestation of BPS and the variation in symptoms according to level of cognitive impairment [66]. There was however a positive association between any antidementia drug and irritability/lability among those with moderate cognitive impairment and in the total study population. This result was also found when acetylcholinesterase inhibitors and memantine were investigated separately. A possible explanation might be that irritability/lability is found to be common at a moderate state of cognitive impairment when clustered as aggressive behavior in another study [66]. The result found in the present study may therefore be considered as an appropriate prescribing pattern since antidementia drug use may decrease the risk of aggressive behavior [43].

Some limitations with the current study should be mentioned. The response rate was 55% which may have introduced responder bias and affected the result in either way, i.e. BPS and psychotropic drug use found in the present study might reflect either a higher or lower prevalence of BPS and drug use in Sweden when the questionnaire was distributed, which also may have affected the associations found in the present study. More specifically how is difficult to know as the reasons for not responding on the questionnaire were not investigated. Nevertheless, this aspect should be taken into consideration when interpreting the result. No causality assessment can be made due to the observational study design. We also lack the temporal aspect of psychotropic drug use and observed BPS which may have affected the result. Drug-use in this study is an estimation from prescription-data and thus we cannot say anything about dose, duration or potential discontinuation of treatment, nor about the indication for drug use. Different BPS may for example coexist leading to associations between drug use and specific symptoms for which the drug was not intended to treat. Moreover, due to lack of information about diagnosis we cannot say anything about the origin of the symptom reported as a BPS, e.g. depression which might be considered as a comorbidity instead. Therefore, it is not possible to state with certainty whether the drug treatment is appropriate or not. Additionally, drug use was defined as having dispensed at least one prescribed drug at least once during a six-month period. This may increase the possibility to identify drugs which were only used occasionally. Consequently, there is a possibility that the identified prevalence might not accurately reflect actual drug use. By grouping the drugs identified on ATC-code level 7, into ATC-code level 4, the drug classes include drugs with different risk-benefit ratio, e.g. melatonin, which was also included in the analyses. This needs to be taken into consideration when interpreting the result. Our results examining the various cognitive subgroups are also purely descriptive since we did not use any statistical analysis comparing if the group differences were statistically significant. Moreover, it is important to consider that our study did not have information about formal MND diagnoses and instead used the Gottfries’ score as a proxy-rating for underlying MND, possibly including persons without MND in our statistical analyses. However, one could argue that this is in fact a strength, since undiagnosed MND appears common in nursing home environments [65]. Strengths of this study that are worth mentioning are the large sample size and that the data-collection was nationwide and randomly selected.

## Conclusions

Except antidementia drugs, for which use was somewhat low, psychotropic drug use is remarkably high among Swedish nursing home residents with BPS and cognitive impairment. Especially antipsychotic and antidepressant drugs warrant concern. Even if several associations indicate a rational prescribing pattern, the results call for further studies examining the treatment of BPS in this vulnerable group of people to ensure compliance with best available treatment practices.

## Supplementary Information

Additional file 1.

Additional file 2.

## Data Availability

The datasets generated and/or analysed during the current study are not publicly available due to ethical standards and legal requirements.

## Abbreviations

ADL: Activities of daily living
ATC: Anatomical Therapeutic Chemical
BPS: Behavioral and psychological symptoms
CI: Confidence interval
IQR: Interquartile range
MMSE: Mini Mental State Examination
MND: Major neurocognitive disorder
NPI-NH: The Neuropsychiatric Inventory - Nursing Home
OR: Odds ratio
SD: Standard deviation
SSRIs: Selective serotonin reuptake inhibitors
SWENIS: The Swedish National Inventory of Care and Health in Residential Aged Care
