# Supplementary material for: Psychotropic drug use among older Swedish nursing home residents with cognitive impairment and behavioral and psychological symptoms: a cross-sectional questionnaire survey
Source: BMC Geriatr. 2026 Jul 22;26:971. doi: 10.1186/s12877-026-08014-4 (PMC13390425; doi:10.1186/s12877-026-08014-4)
Supplement: Supplementary file 1 — Additional file 1. [file 12877_2026_8014_MOESM1_ESM.pdf]

Psychotropic drug use among older Swedish nursing home residents with cognitive impairment and behavioral and psychological symptoms: a cross-sectional questionnaire study

Additional file 1

Sönnerstam Eva[a], Andersson Tomas[b], Backman Annica[c], Sköldunger Anders[c], Edvardsson David[d], Gustafsson Maria[a], Lövheim Hugo[b]

[a] Department of Medical and Translational Biology, hus H, Johan Bures väg 12, Biologihuset, Umeå university, 901 87 Umeå, Sweden. E-mail addresses: eva.sonnerstam@umu.se, maria.gustafsson@umu.se

[b] Department of Community Medicine and Rehabilitation, Umeå University, 901 87 Umeå, Sweden. E-mail addresses: tomas.n.andersson@regionvasterbotten.se, hugo.lovheim@umu.se

[c] Department of Nursing, Umeå University, 901 87 Umeå, Sweden. E-mail addresses: annica.backman@umu.se, anders.sjoldunger@umu.se

[d] Department of Nursing, Swinburne University of Technology, Melbourne, Australia. Sahlgrenska Academy, Institute of Health and Care Sciences, University of Gothenburg, Sweden. E-mail address: dedvardsson@swin.edu.au

BMC Geriatrics

Corresponding author:

Eva Sönnerstam, eva.sonnerstam@umu.se

Visualization of data collection

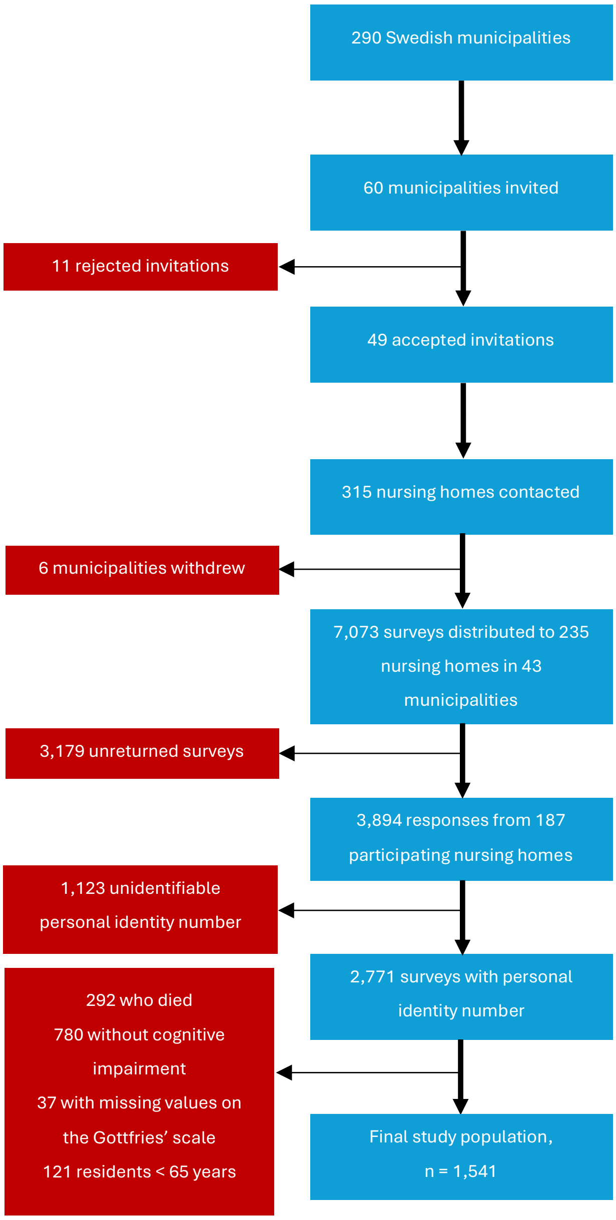

Fig. A.1; Flowchart. Data collection flow chart

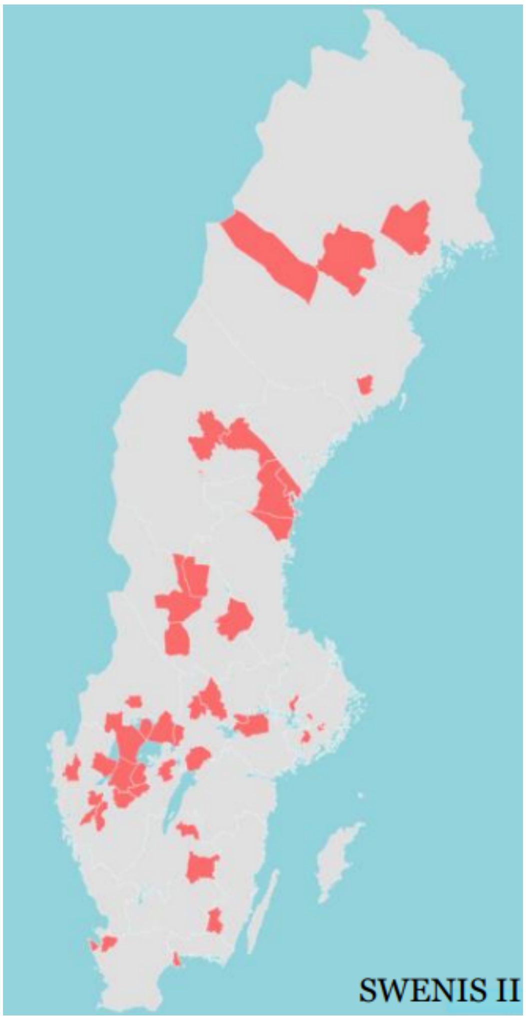

Fig. A.2; Participating municipalities. The geographical distribution of the participating municipalities (marked with red) in SWENIS II.
