# Supplementary material for: Psychotropic drug use among older Swedish nursing home residents with cognitive impairment and behavioral and psychological symptoms: a cross-sectional questionnaire survey
Source: BMC Geriatr. 2026 Jul 22;26:971. doi: 10.1186/s12877-026-08014-4 (PMC13390425; doi:10.1186/s12877-026-08014-4)
Supplement: Supplementary file 2 — Additional file 2. [file 12877_2026_8014_MOESM2_ESM.pdf]

Psychotropic drug use among older Swedish nursing home residents with cognitive impairment and behavioral and psychological symptoms: a cross-sectional questionnaire study

Additional file 2

Sönnerstam Eva[a], Andersson Tomas[b], Backman Annica[c], Sköldunger Anders[c], Edvardsson David[d], Gustafsson Maria[a], Lövheim Hugo[b]

[a] Department of Medical and Translational Biology, hus H, Johan Bures väg 12, Biologihuset, Umeå university, 901 87 Umeå, Sweden. E-mail addresses: eva.sonnerstam@umu.se, maria.gustafsson@umu.se

[b] Department of Community Medicine and Rehabilitation, Umeå University, 901 87 Umeå, Sweden. E-mail addresses: tomas.n.andersson@regionvasterbotten.se, hugo.lovheim@umu.se

[c] Department of Nursing, Umeå University, 901 87 Umeå, Sweden. E-mail addresses: annica.backman@umu.se, anders.sjoldunger@umu.se

[d] Department of Nursing, Swinburne University of Technology, Melbourne, Australia. Sahlgrenska Academy, Institute of Health and Care Sciences, University of Gothenburg, Sweden. E-mail address: dedvardsson@swin.edu.au

BMC Geriatrics

Corresponding author:

Eva Sönnerstam, eva.sonnerstam@umu.se

Table A.1; Significant associations between BPS and basic characteristics, and psychotropic drug use among residents with mild cognitive impairment. Results are presented as odds-ratios (OR) with the 95%-confidence interval (CI).

	**Anti-psychotics**	**Anxiolytics**	**Hypnotics and sedatives**	**Anti-depressants**	**Antidementia drugs**	**Acetyl-cholinesterase inhibitors**	**Memantine**
Age	0.96 (0.93-0.98)	0.981 (0.977-0.986)	0.984 (0.982-0.987)			0.97 (0.96-0.98)	
Sex							
Women				1.45 (1.14-1.83)			
Men				Ref			
Gottfries' score (16-23)					0.91 (0.89-0.93)		0.89 (0.86-0.91)
Katz' ADL (0-6)		1.11 (1.01-1.21)			1.25 (1.14-1.38)	1.35 (1.20-1.52)	1.13 (1.01-1.27)
Delusions					1.09 (1.01-1.17)		1.13 (1.05-1.22)
Halluci-nations						1.11 (1.01-1.21)	
Depression/ Dysphoria				1.17 (1.08-1.28)			
Anxiety		1.21 (1.11-1.31)					
Aberrant motor behavior		0.90 (0.82-0.98)					
Sleep and Nighttime Behavior Disorders		1.18 (1.08-1.28)	1.19 (1.09-1.29)				
Appetite and Eating Disorders							0.82 (0.69-0.98)

ADL, Activities of Daily Living
Ref, Reference

Table A.2; Significant associations between BPS and basic characteristics, and psychotropic drug use among residents with moderate cognitive impairment. Results are presented as odds-ratios (OR) with the 95%-confidence interval (CI).

	**Anti-psychotics**	**Anxiolytics**	**Hypnotics and sedatives**	**Anti-depressants**	**Antidementia drugs**	**Acetyl-cholinesterase inhibitors**	**Memantine**
Age	0.980 (0.977-0.983)	0.987 (0.984-0.990)	0.979 (0.976-0.983)		0.985 (0.981-0.988)	0.975 0.970-0.980)	0.980 (0.975-0.984)
Katz' ADL (0-6)					1.21 (1.10-1.32)	1.25 (1.12-1.40)	1.15 (1.04-1.28)
Halluci-nations			1.08 (1.002-1.16)				
Agitation/ Aggression	1.09 (1.03-1.16)						
Depression/ Dysphoria		1.10 (1.003-1.20)		1.17 (1.08-1.26)			
Anxiety	1.07 (1.01-1.14)	1.09 (1.01-1.17)	1.12 (1.03-1.22)				
Elation/ Euphoria							1.14 (1.002-1.29)
Irritability/ Lability					1.15 (1.08-1.22)	1.12 (1.05-1.20)	1.08 (1.02-1.15)
Sleep and Nighttime Behavior Disorders		1.19 (1.10-1.29)	1.21 (1.12-1.31)			0.89 (0.80-0.98)	

ADL, Activities of Daily Living

Table A.3; Significant associations between BPS and basic characteristics, and psychotropic drug use among residents with severe cognitive impairment. Results are presented as odds-ratios (OR) with the 95%-confidence interval (CI).

	**Anti-psychotics**	**Anxiolytics**	**Hypnotics and sedatives**	**Anti-depressants**	**Antidementia drugs**	**Acetyl-cholinesterase inhibitors**	**Memantine**
Age	0.975 (0.967-0.983)	0.982 (0.977-0.986)	0.975 (0.970-0.980)		0.991 (0.987-0.995)	0.974 (0.968-0.979)	0.988 (0.984-0.993)
Gottfries' score (0-7)	1.15 (1.01-1.32)						
Katz' ADL (0-6)				1.21 (1.002-1.47)	1.27 (1.07-1.51)		1.32 (1.10-1.57)
Delusions					0.90 (0.81-0.997)		0.84 (0.74-0.96)
Halluci-nations							1.14 (1.03-1.27)
Agitation/ Aggression			1.12 (1.01-1.23)				
Depression/ Dysphoria			1.16 (1.001-1.34)				
Anxiety		1.12 (1.02-1.21)					
Apathy/ Indifference		1.09 (1.007-1.18)					
Aberrant Motor Behavior	1.13 (1.05-1.21)	1.19 (1.10-1.29)	1.13 (1.03-1.23)				
Appetite and Eating Disorders					0.82 (0.70-0.98)		0.83 (0.70-0.996)

ADL, Activities of Daily Living
